# Deregulation of COMMD1 Is Associated with Poor Prognosis in Diffuse Large B-cell Lymphoma

**DOI:** 10.1371/journal.pone.0091031

**Published:** 2014-03-13

**Authors:** Minna Taskinen, Riku Louhimo, Satu Koivula, Ping Chen, Ville Rantanen, Harald Holte, Jan Delabie, Marja-Liisa Karjalainen-Lindsberg, Magnus Björkholm, Øystein Fluge, Lars Møller Pedersen, Karin Fjordén, Mats Jerkeman, Mikael Eriksson, Sampsa Hautaniemi, Sirpa Leppä

**Affiliations:** 1 Department of Oncology, Helsinki University Central Hospital, Helsinki, Finland; 2 Genome Scale Biology Program, University of Helsinki, Helsinki, Finland; 3 Department of Oncology, Oslo University Hospital, Oslo, Norway; 4 Department of Pathology, Oslo University Hospital, Oslo, Norway; 5 Department of Pathology, Haartman Institute, University of Helsinki, Helsinki, Finland; 6 Department of Medicine, Karolinska University Hospital, Stockholm, Sweden; 7 Department of Oncology and Medical Physics, Haukeland University Hospital, Bergen, Norway; 8 Department of Hematology, Odense University Hospital, Odense, Denmark; 9 Department of Oncology, Lund University Hospital, Lund, Sweden; The University of North Carolina at Chapel Hill, United States of America

## Abstract

**Background:**

Despite improved survival for the patients with diffuse large B-cell lymphoma (DLBCL), the prognosis after relapse is poor. The aim was to identify molecular events that contribute to relapse and treatment resistance in DLBCL.

**Methods:**

We analysed 51 prospectively collected pretreatment tumour samples from clinically high risk patients treated in a Nordic phase II study with dose-dense chemoimmunotherapy and central nervous system prophylaxis with high resolution array comparative genomic hybridization (aCGH) and gene expression microarrays. Major finding was validated at the protein level immunohistochemically in a trial specific tissue microarray series of 70, and in an independent validation series of 146 patients.

**Results:**

We identified 31 genes whose expression changes were strongly associated with copy number aberrations. In addition, gains of chromosomes 2p15 and 18q12.2 were associated with unfavourable survival. The 2p15 aberration harboured *COMMD1* gene, whose expression had a significant adverse prognostic impact on survival. Immunohistochemical analysis of COMMD1 expression in two series confirmed the association of COMMD1 expression with poor prognosis.

**Conclusion:**

COMMD1 is a potential novel prognostic factor in DLBCLs. The results highlight the value of integrated comprehensive analysis to identify prognostic markers and genetic driver events not previously implicated in DLBCL.

**Trial Registration:**

ClinicalTrials.gov NCT01502982

## Introduction

Diffuse large B-cell lymphoma (DLBCL) is the most common lymphoid neoplasm. It is an aggressive lymphoma entity, and only 50% of patients can be cured with anthracycline-based CHOP (cyclophosphamide, doxorubicin, vincristine, and prednisone) or CHOP-like chemotherapy. However, following the addition of rituximab or etoposide to CHOP, or the administration of CHOP dose-densely at two-week intervals (CHOP-14), response rates and survival have significantly improved [1]–[5]. Despite these advances, 20–30% of patients experience disease relapses or have primary refractory disease. Such patients could benefit from alternative therapies if their clinical outcome could be more accurately predicted at the time of diagnosis. Therefore, the identification of biological prognostic factors that could identify high-risk DLBCL patients is a priority.

Genome-wide molecular profiling has revealed a high degree of complexity in DLBCL,and significantly accelerated the understanding of oncogenic mechanisms in lymphomagenesis [6], [7]. On the basis of gene expression profiling (GEP), DLBCL is classified into distinct molecular subtypes [8]–[11]. Three major DLBCL entities, showing germinal center B-cell (GCB), activated B-cell (ABC)-like, and primary mediastinal B-cell lymphoma signatures have been recognized. Many oncogenic mechanisms distinguish GCB and ABC subtypes. For example, chromosomal translocations involving *BCL2* and the c-*REL* locus amplification on chromosome 2p occur predominantly in the GCB DLBCLs [10], [12]. In contrast, ABC DLBCLs are characterized by transcriptional overexpression of *BCL2* and a constitutive activation of the NF-κB signaling pathway [10], [13]. According to the gene expression based classification, the patients in the molecular subgroups also have different outcomes in response to chemo- and chemoimmunotherapy [9], [10].

Over the past few years, progress in molecular genetics and sequencing technologies has also revealed several previously unrecognized genetic lesions and pathways that are involved in DLBCL [14]–[17]. For example, recurrent mutations inactivating histone and/or chromatin modifying genes, and genes involved in immune recognition have been identified. However, despite the rapidly growing number of genetic aberrations reported in DLBCL, association of these findings with treatment outcome remains to be shown.

We have integrated the information from high-resolution gene copy number and expression microarrays to identify the most likely “driver gene” candidates associated with DNA copy number aberrations (CNAs) and poor prognosis in DLBCL. Importantly, with our cohort of high-risk DLBCL patients treated homogenously in a phase II study with dose-dense chemoimmunotherapy and systemic CNS prophylaxis, we were able to identify a genomic region harbouring a gene that has a survival effect and thus is a candidate for a novel molecular marker for poor prognosis.

## Materials and Methods

### Ethics Statement

Written informed consent was obtained prior to treatment and sampling from all patients included in the NLG-LBC-04 study. Clinical protocol and sampling were approved in the participating countries at the national level by Regional Committee on Health Research Ethics in Glostrup, Denmark, the Hospital District of Helsinki and Uusimaa Regional Committee on Medical Research Ethics in Finland, Oslo Regional Committee for Medical and Health Research Ethics in Norway, and Lund Regional Ethics Committee in Sweden. The trial was registered at ClinicalTrials.gov, number NCT01502982. For the retrospectively collected validation cohort, approval was obtained from the National Authority for Medicolegal Affairs, Finland and Helsinki University Central Hospital, Finland.

### Patients

The prospectively collected screening (aCGH) and tissue microarray (TMA) cohorts consisted of DLBCL patients who were less than 65 years old and had primary high-risk (age-adjusted International Prognostic Index (aaIPI) score 2–3) disease. They were treated in the Nordic phase II NLG-LBC-04 protocol with six courses of R-CHOEP14 (rituximab, cyclophosphamide, doxorubicin, vincristine, etoposide, and prednisone supported with G-CSF) followed by systemic CNS prophylaxis with one course of high-dose methotrexate and one course of high-dose cytarabine [18]. The original clinical study included 156 eligible patients. Histological diagnosis was established from surgical or needle biopsy of the pretreatment tumour tissue by local pathologists according to current criteria of the World Health Organization classification [19], and subsequently reviewed by expert hematopathologists on a national basis. The patient selection for this molecular study was based on availability of fresh frozen tissue containing adequate material for DNA extraction and aGCH (screening cohort; n = 51), and for RNA extraction and gene expression profiling (n = 38). The infiltration of lymphoma cells in the tissue was assessed from frozen tissue section using hematoxylin eosin and toluidine blue stainings. Formalin-fixed paraffin-embedded (FFPE) tissue containing adequate material was used for the preparation of TMAs (TMA cohort; n = 70).

To validate the findings, an independent series of 146 primary DLBCL patients treated with chemoimmunotherapy at the Helsinki University Central Hospital between 2001 and 2010 was used. The patients were treated with R-CHOP (n = 126), R-CHOEP (*n* = 11) or other regimen (*n* = 9). The cases were selected based on the availability of FFPE tissue and clinical information.

### Samples

RNA and DNA were extracted with Qiaqen AllPrep DNA/RNA/Protein Mini kit. CNAs were analysed from the DNA of 51 tumour samples hybridized onto Agilent Human (4×) 180 K CGH arrays. Tumour samples from 38 patients were eligible for mRNA analyses using Affymetrix Human Exon 1.0 ST arrays. All hybridizations were performed at the Biomedicum Genomics (University of Helsinki) according to manufacturer’s instructions. Hybridization protocols and raw expression microarray data are available at ArrayExpress archive http://www.ebi.ac.uk/arrayexpress/experiments/E-MEXP-3488 and http://www.ebi.ac.uk/arrayexpress/experiments/E-MEXP-3463. All tissue samples were collected before treatment.

### qRT-PCR

Expression of the *COMMD1* (Hs04190004_m1) and *XPO1* (Hs00418963_m1) genes were validated by quantitative real-time polymerase chain reaction (qRT-PCR) using TaqMan Gene Expression Assays (Assays-On-Demand, Applied Biosystems) and the ABI Prism 7500 Fast Sequence Detection System (Applied Biosystems) for 24 available tumour samples. Normalization for the quantity of DNA was done by performing simultaneous qRT-PCR for *GAPDH* (TaqMan Pre-Developed Assay Reagents, Applied Biosystems). Each assay was determined by a comparative cycle threshold method, using the arithmetic formula provided by the manufacturer. All assays were performed in triplicate.

### Subgroup Classification by Gene Expression Profiling

Samples involved in exon array analysis were divided into germinal centre B-cell (GCB) and non-GCB subgroups by gene expression profiling (GEP). Briefly, we utilized the log ratios of 44 genes from the gene expression panel by Wright et al. [20] and agglomerative hierarchical clustering (complete linkage) to divide samples into two subgroups. The *IGHM* gene was not on our array and was subsequently dropped from the analysis. Following the Wright classification [20], samples in one main branch of the resulting cluster tree were categorized as GCB and samples in the other branch as non-GCB DLBCLs. In addition, all samples were classified into GCB and non-GCB phenotypes immunohistochemically (IHC) according to Hans algorithm [21].

### Immunohistochemical Analyses of COMMD1

IHC stainings were performed on FFPE tissue sections on TMA slides containing 2–4 tissue cores/patient, with a core diameter of 1 mm (TMA cohort), or whole tissue sections (independent validation cohort). After deparaffinization, heat-induced epitope retrieval (121°C, 3 min), and blocking of endogenous peroxidase, the slides were incubated with anti-COMMD1 antibody (1∶200, Sigma-Aldrich, Prestige Antibodies) at 4°C overnight. Staining was completed with Vectastain ABC kit reagents (Vector Laboratories) according to the manufacturer’s instructions, and slides were counterstained with hematoxylin.

To score the stainings, COMMD1-positivity was evaluated from one to three high-power fields (hpf; x630 magnification) with the Leica DM LB bright-field microscope (Leica Microsystems GmbH) and a camera attached to it (Olympus DP50, InStudio 1.0.1 Software). The most representative areas with intense staining pattern were first selected with low magnification and further digitized with hpf, resulting in microscopic images with area size of 0.02 mm^2^. Images were subsequently scored using computerized image analysis system [22]. All scorings were performed blindly.

Quantitative image analyses were performed using Anduril [22]. The colour space of each image was categorized to four expected representative colour classes: Brown, blue, white and background. The background class included faint brown and blue colours considered to be unspecific staining. The colour values were selected by pointing at 15 example colours for each class. All images were subjugated to use the same class specification. Each pixel was assigned to a class by finding the nearest example colour value, and a final staining coverage calculated from the area of each class present in each image.

### Data Analysis

In copy number profiling the aCGH data were first normalized with locally weighted scatterplot smoothing (LOWESS) normalization (four iterations, 30% window) and the data were denoised with circular binary segmentation (*p*<0.05, split undo = none) [23]. The background noise level of copy number arrays was estimated by calculating the median probe signal of all arrays. An aberration was called significant if it was two standard deviations apart from the median. We identified minimal common CNA regions in which a CNA overlapped in 10% of the samples with both transcriptome and copy number data, and subsequent analysis were restricted to these regions. The Database of Genomic Variants (DGV, version 10, Nov 2010) [24] was used to determine locus specific copy number variants (CNVs). In short, for each gene in our CNA regions, the number of overlapping CNVs in the DGV according to genomic locus was counted. Genes with more than 10 CNVs were excluded from the subsequent analyses. Exon array expression data were normalized and transformed to gene expression level data by the Multiple Exon Array Preprocessing (MEAP) algorithm [25].

In order to find genes with a significant association of expression and CNA, all genes in the expression data, which were located in a minimal common CNA region, were first matched with their respective segmented copy number values. The samples were split into two groups based on their CNA status, separately for gains and losses, and a signal-to-noise statistic on the expression of each gene was calculated [26]. A *p*-value for each signal-to-noise score was calculated with a permutation test. Gain or deletion was associated with expression for genes that displayed up- or downregulation in CNA samples but stable expression in non-CNA samples (*p*<0.05). Moreover, only genes that exhibited CNA in at least three patients were analysed.

A Chi square test was performed to evaluate the differences in the frequency for the prognostic factors. Categorical data were compared using the Fisher’s exact test (two-sided). Pearson correlation coefficient was calculated to evaluate the correlation between the expression values from microarray and qRT-PCR analyses. All genes with altered copy number levels in at least five samples were analysed for patient survival. Survival curves with corresponding *p*-values were calculated using Kaplan-Meier analysis with the log-rank test. Receiver operating characteristic (ROC) curve analysis was used to determine the ideal cutoff values for survival outcomes. Univariate analyses were performed according to the Cox proportional hazards regression model. The progression-free survival (PFS) was calculated as the period between the dates of registration and lymphoma progression or relapse. Otherwise, the patients were censored at the last date of follow-up. Patients in remission were censored at the last date they were known to be alive. Patients who died due to causes other than lymphoma were censored at the date of death. Overall survival (OS) was calculated as a period between registration date and date of death. Surviving patients were censored at the last date they were known to be alive. Lymphoma-specific OS was calculated as a period between registration date and the date of death due to lymphoma. P-values less than 0.05 were considered to indicate statistical significance. Data analyses were done with the computational framework Anduril [22], which is designed for systematic integration, analysis and result interpretation of large-scale molecular data, and with IBM SPSS Statistics 20.0.

## Results

### Clinical Characteristics of the Screening Cohort

The baseline characteristics of the screening (aCGH) cohort of 51 patients treated in the NLG-LBC-04 protocol [18] are shown in Table 1. Median age of the patients was 55 years (range, 20–65 years). The overall response rate (ORR; complete response (CR)+partial response (PR)) in this study population was 98%. Median follow-up of the patient cohort was 55 months (range 31–101 months), 15 patients had relapsed, three experienced CNS relapse and 12 had died. Five of the deaths were not lymphoma-related. Predicted 5-year PFS was 69%, lymphoma-specific OS 85%, and OS 76%.

**Table 1:** Patient characteristics in the screening cohort.

	All n (%)	51 (100)
Gender	Female	19 (37)
	Male	32 (63)
Age	Median (range)	55 (20–65)
	<60	35 (69)
	60–65	16 (31)
	>65	0 (0)
Histology	GCB	27 (53)
	Non-GCB	20 (39)
	Other/Unclassified	4 (8)
Performance status	0–1	33 (65)
	2–3	18 (35)
B-symptoms		31 (61)
Elevated LDH		49 (96)
Stage	I–II	1 (3)
	III–IV	37 (97)
aaIPI	0	0 (0)
	1	0 (0)
	2	36 (71)
	3	15 (29)
mRNA analysis		38 (75)

### Gene Copy Number Aberrations

Genome-wide copy number analysis of 51 lymphoma samples with aCGH revealed several gains and losses. All patients had at least one abnormality with an average number of 17.5±9.8 CNAs per patient. The most frequently (≥10%) altered regions as well as their frequencies and possible target genes are shown in Table 2. Some samples exhibited narrower alterations than others, which caused the small variation in the CNA frequencies. Of the recurrent CNAs previously reported in DLBCL [27]–[30], gains in 3q, 7q22.1, and 19p13, and loss in 6q were observed in 9.8% of the patients.

**Table 2:** Genome-wide overview of recurrent gains and losses.

Band	Gain Freq %	Loss Freq %	Position (Mb)	Possible target genes
1q24.2	12	8	167.69–167.76	MPZL1
1q44	20	6	247.00–247.10	AHCTF1
2p16.1–p15	12	NA	60.68–63.27	BCL11A, PAPOLG, REL, PUS10, PEX13, KIAA1841, AHSA2, USP34, XPO1, FAM161A, CCT4, COMMD1, B3GNT2, TMEM17, EHBP1
9p21.3	NA	14–18	21.80–22.01	MTAP, CDKN2A, C9orf53, CDKN2B
14q11.2	2	12	22.938–22.939	TRDV3
18q12.2	12	NA	34.82–35.15	CELF4
18q21.1	12	4	44.06–44.34	LOXHD1, ST8SIA5
18q23	12–16	4	77.62–77.71	KCNG2, PQLC1
20q11.22	16	NA	33.13–33.15	MAP1LC3A

The association of CNAs with molecular subgroups is summarized in Table S1 in File S1. Notably, GCB type DLBCL was characterized by more frequent gain of 2p15 and 2p16.1 including the well-known proto-oncogenes *REL* and *BCL11A* as compared to non-GCB DLBCL (15–19% vs. 5%, *p* = ns). Instead, the most frequently altered genomic regions in the non-GCB DLBCL subgroup in comparison to GCB DLBCL patients were gains of 18q12.2 (20% vs. 4%, *p* = ns) and 18q23 (20% vs. 4–7%, *p* = ns), and loss of 9p21.3 (30–35% vs. 7%, *p* = 0.026). Of the other genomic imbalances (<10% of all patients), only the gain of 18q21.2–33 in the non-GCB subgroup was significantly more frequent when compared to GCB subgroup (20% vs. 0%, *p* = 0.027).

### Copy Number Associated Gene Expression Changes

To identify genes with altered expression due to large genomic aberrations, we combined the CNA and gene expression data obtained from 38 patients for whom both data sets were available. Our analysis showed that copy number gains and losses of 31 genes were associated with a simultaneous and significant increase or decrease in gene expression. The majority (*n* = 29) of the genes were over-expressed due to copy number gains in chromosomes 2 (2p15 and 2p16.1) and 18 (18q21.2, 18q21.31–33, 18q23). In contrast, two genes were suppressed and located in regions of copy number losses at 9p21.3. The aforementioned CNA areas and target genes are presented in more detail in Table S2 in File S1 and at http://csbi.ltdk.helsinki.fi/pub/lymphoma. As an example, the patients with 2p15 amplification had elevated COMMD1 expression with a p-value and fdr <0.001.

### Prognostic Significance of Chromosomal Alterations

**Figure 1:**
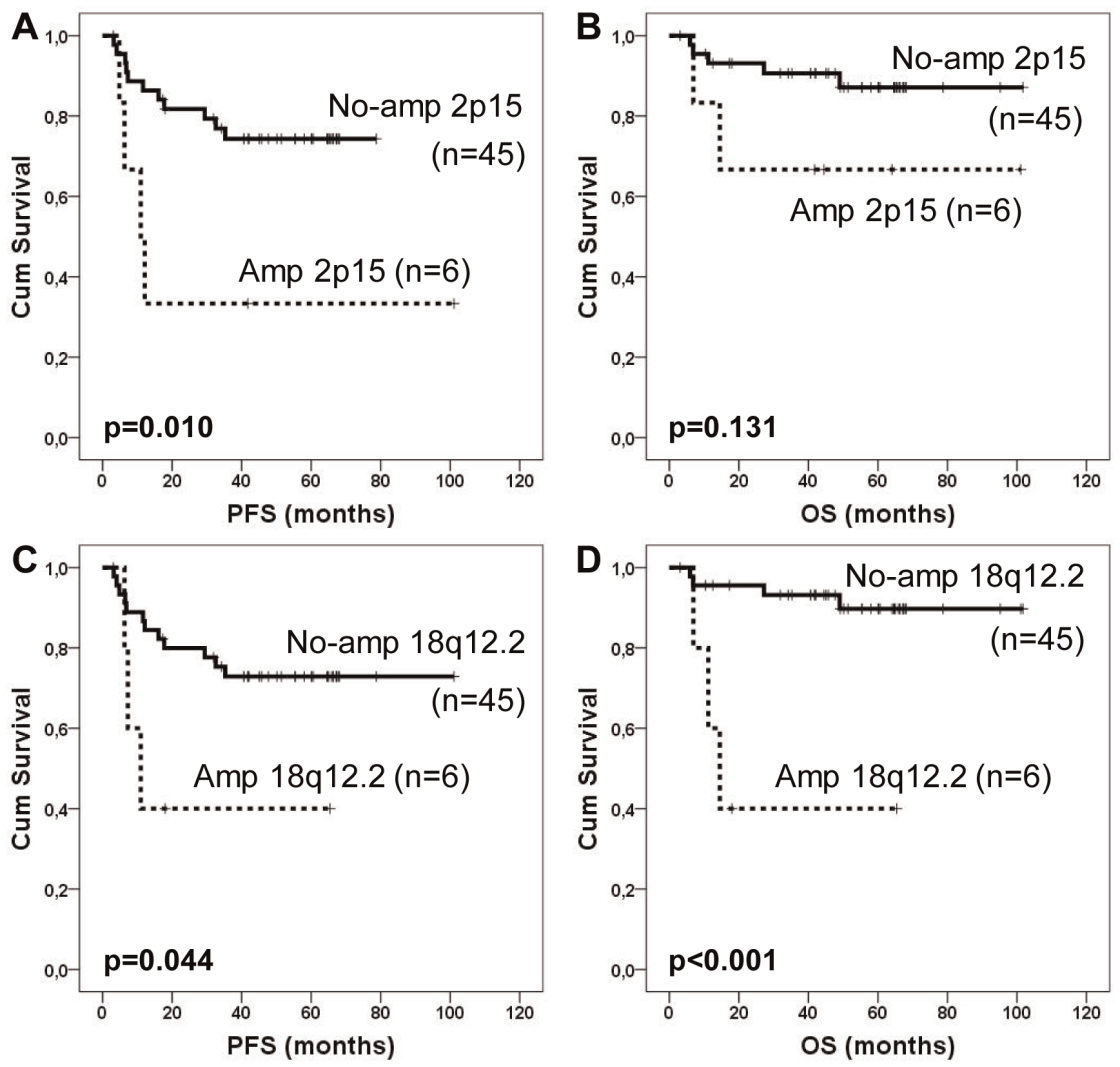
Survival of DLBCL patients according to genomic aberrations. PFS (A and C) and lymphoma-associated OS (B and D) rates according to indicated genomic aberration.

**Figure 2:**
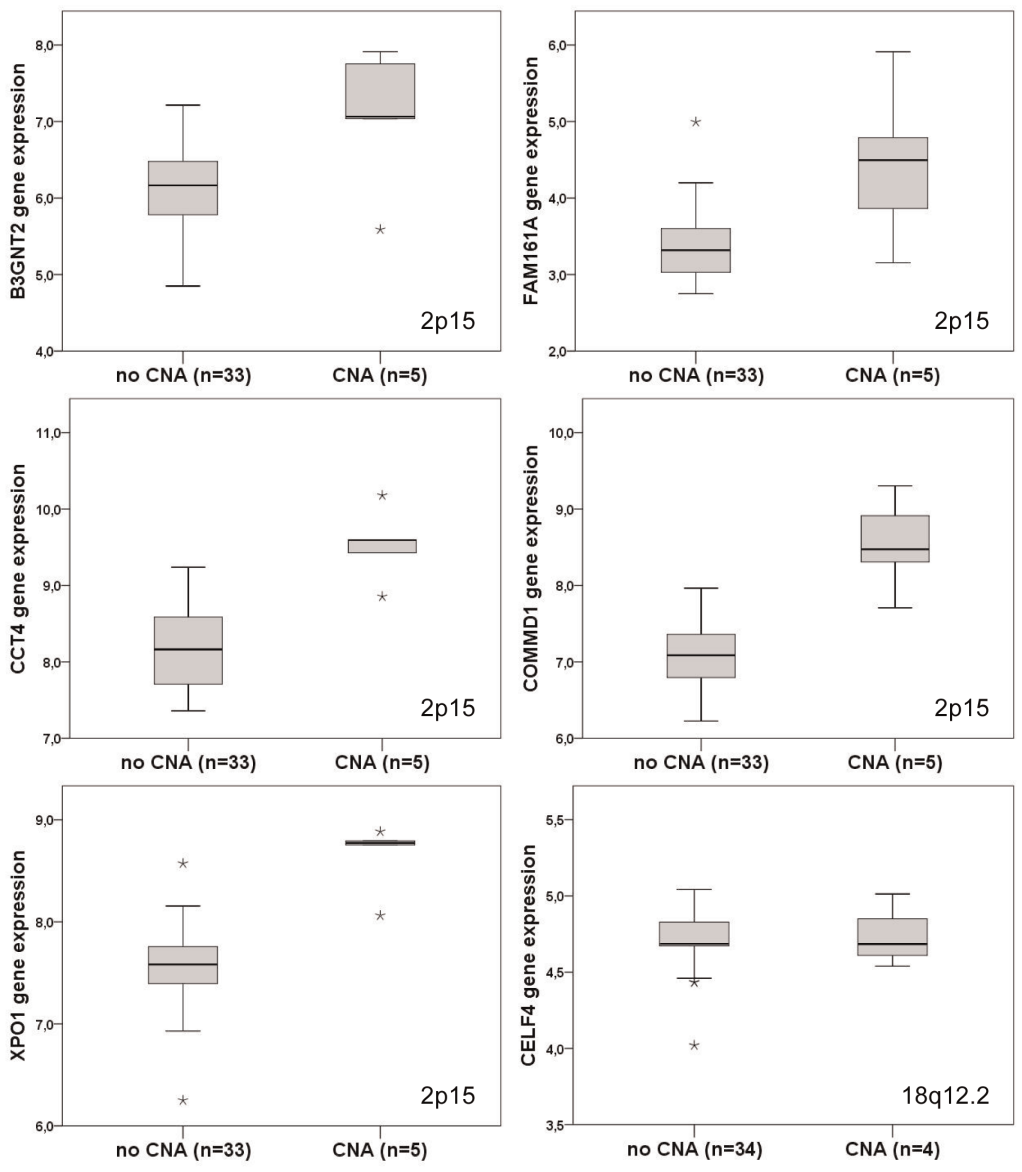
Expression of genes associated with amplifications in 2p15 and 18q12.2 locuses. Boxes contain expression values between the 25^th^ and 75^th^ percentile in the tumour subgroup. The extremes denoted by asterisks represent maximum and minimum expression values.

In the whole series of 51 patients with the aGCH data, we found two chromosomal regions with genomic alterations associated with PFS and lymphoma-specific OS. Patients with amplification in chromosome 2p15 (*n* = 6; 12% of all patients) had inferior PFS in comparison to patients without this gain (*p* = 0.010; Figure 1A). In addition a non-significant difference towards poor lymphoma-specific OS was observed (*p* = 0.131; Figure 1B). Similarly, patients with amplification in 18q12.2 (*n* = 6) had worse PFS (*p* = 0.044) and lymphoma-specific OS (*p*<0.001) than patients without this gain (Figures 1C and 1D). The survival associated gain in 2p15 contained the genes *B3GNT2* (UDP-GlcNAc:betaGal beta-1,3-N-acetylglucosaminyltransferase 2), *FAM161A* (family with sequence similarity 161, member A), *CCT4* (chaperonin containing TCP1 subunit 4), *COMMD1* (copper metabolism (Murr1) domain containing 1), and *XPO1* (exportin 1), and the amplification was associated with their over-expression (Figure 2). Association of *COMMD1* and *XPO1* over-expression with 2p15 amplification was further confirmed by qRT-PCR (Figure S1 in File S1). The amplification in 18q12.2 contained the *CELF4* gene but we found no correlation between the gain and *CELF4* gene expression (Figure 2).

Consistent with previous studies on lymphomas [27], [28], [31], we identified *REL* and *BCL11A* located at 2p16.1, and *BCL2* at 18q21.3 being among the genes, whose expression was linked with copy number gains (Table S2 in File S1). *CDKN2A* and *MTAP* genes, which have also been described in lymphomas, specifically in the chemoresistant and ABC type DLBCLs [27], [28], [30], [32], [33]}, were in turn located in the regions of copy number losses at 9p21.3. However, these genomic alterations were not associated with survival in our study population.

**Figure 3:**
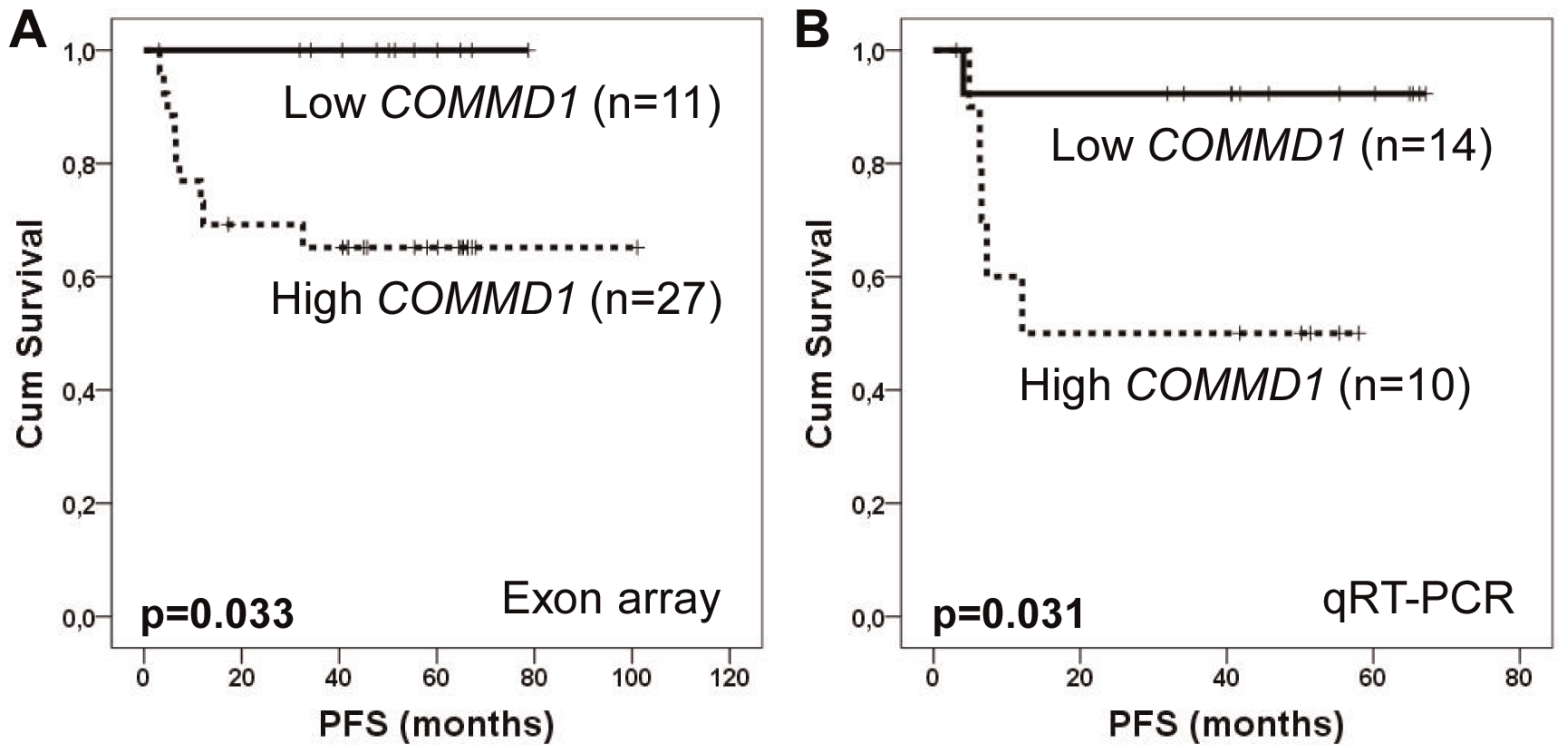
PFS according to *COMMD1* expression. A. PFS according to exon array based *COMMD1* expression values. B. PFS according to quantitative PCR analysis based *COMMD1* expression values. In both A and B, the ideal cutoff values have been calculated using ROC curve analyses. In A the estimated area under the curve (AUC) was 0.717 (*p* = 0.063, 95% CI 0.531–0.903). In B the AUC was 0.759 (*p* = 0.062, 95% CI 0.468–1.000).

To further identify biomarker candidates located in the survival associated 2p15 amplification locus, we performed survival analysis for five genes whose expression values correlated with amplification. Using gene expression values as continuous variables in Cox univariate analyses, only *COMMD1* expression was identified to have prognostic impact on PFS (*p* = 0.037). When Kaplan-Meier analysis was performed, patients with high *COMMD1* expression had significantly inferior PFS as compared to patients with low expression (5-year PFS 65% vs. 100%, *p* = 0.033; Figure 3A). Association of *COMMD1* expression with the survival was further validated using qRT-PCR and Cox univariate analysis with continuous variables (*p* = 0.009) and Kaplan-Meier analysis with categorical data (*p* = 0.031, Figure 3B). In comparison, the expression of *XPO1*, another selected gene for qRT-PCR validation, was not significantly associated with survival (*p* = 0.345). Correlation coefficients between the expression arrays and qRT-PCR were 0.641 (*p*<0.001) for *COMMD1* and 0.494 (*p* = 0.037) for *XPO1*.

### COMMD1 Protein Expression is Associated with Outcome

**Figure 4:**
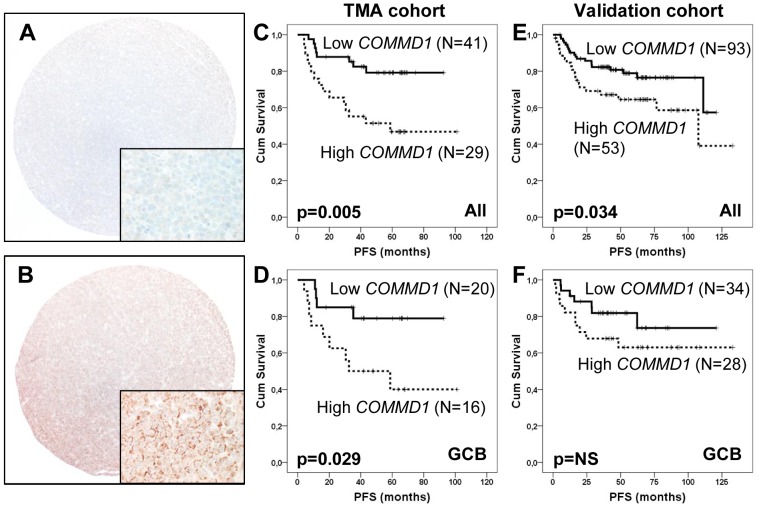
COMMD1 protein expression and outcome. Representative examples of low (A) and high (B) expression levels of COMMD1 in FFPE DLBCL tissue (original magnifications 100×, and 400×). C–D. Outcome according to COMMD1 expression in the trial specific TMA cohort. PFS in in the whole TMA cohort (C) and in the GCB subgroup (D). E–F. Outcome according COMMD1 expression in the validation cohort. PFS in in the whole validation cohort (E) and in the GCB subgroup (F). The cutoff point (staining coverage of 8.9%) for survival outcomes (COMMD1 low vs high) was selected by the ROC curve analysis in the training set, and then applied also to validation cohort.

Considering that our multi-level analysis revealed *COMMD1* to be amplified, over-expressed and survival associated gene in DLBCL, we extended COMMD1 analyses to the protein level. IHC stainings were performed on a TMA consisting of 70 lymphoma samples from the patients treated in the NLG-LBC-04 protocol (Table 3). Overall, intensity of COMMD1 positivity was highly variable (Figure 4A–B). COMMD1 immunoreactivity was primarily localized as perinuclear, granular, cytoplasmic pattern in lymphoma cells (Figure 4B), but also in endothelial cells and macrophages with more uniform cytoplasmic staining pattern.

**Table 3:** Baseline characteristics according to COMMD1 expression in TMA and validation cohorts.

		TMA cohort(n = 70)			Validation cohort (n = 146)		
	Number of patients	COMMD1 low	COMMD1 high	p	COMMD1 low	COMMD1 high	p
	All n (%)	41 (100)	29 (100)		93 (100)	53 (100)	
Gender	Female	13 (32)	11 (38)	0.618	34 (37)	26 (49)	0.163
	Male	28 (68)	18 (62)		59 (63)	27 (51)	
Subtype	GCB	20 (49)	16 (55)	1.000	34 (37)	28 (53)	0.115
	Non-GCB	10 (24)	9 (31)		52 (56)	24 (45)	
	FL grade 3B	4 (10)	1 (3)		2 (2)	1 (2)	
	Other/unknown	7 (17)	3 (10)		5 (5)	0 (0)	
Age	<60	27 (66)	22 (76)	0.434	40 (43)	22 (42)	1.000
	60–65	14 (34)	7 (24)		10 (11)	8 (15)	
	>65	0 (0)	0 (0)		43 (46)	23 (43)	
B-symptoms		24 (59)	13 (45)	0.333	23 (27)	13 (27)	1.000
Elevated LDH		38 (93)	29 (100)	0.261	57 (62)	41 (77)	0.067
Performance status	0–1	32 (78)	13 (45)	0.006	73 (79)	41 (77)	1.000
	2–4	9 (22)	16 (55)		20 (21)	12(23)	
Stage	I-II	0 (0)	1 (3)	0.414	46 (50)	21 (40)	0.301
	III-IV	41 (100)	28 (97)		47 (50)	32 (60)	
IPI	0–2	17 (41)	9 (31)	0.455	57 (65)	30 (59)	0.586
	3–5	24(59)	20 (69)		31 (35)	21 (41)	

The prognostic significance of COMMD1 expression and correlation with mRNA data were assessed by computerized image analysis of COMMD1 positivity in the tumour tissue. In the univariate analysis the increasing COMMD1 positivity was an adverse prognostic factor for PFS (*p* = 0.003). The cutoff point for survival outcomes was selected by ROC curve analysis, resulting in a staining coverage of 8.9% being the most discriminative value (median 7.3%, range 0–24%), with an area under the curve (AUC) value of 0.663 (95% CI 0.516–0.810, *p* = 0.027). In Kaplan-Meier analyses, the patients with high COMMD1 expression had a significantly worse PFS and a trend towards adverse lymphoma associated OS in comparison to the remaining patients with lower COMMD1 expression (5-year PFS 47% vs. 79%, *p* = 0.005 (Figure 4C) and 5-year OS 75% vs. 90%, *p* = 0.081). According to COMMD1 expression, the relative risk of relapse was 3.2 (95% CI 1.361–7.584, p = 0.008) and death 2.825 (95% CI 0.837–9.536, p = 0.094). In multivariate analysis with aaIPI, COMMD1 expression retained its prognostic value on PFS (RR 2.996; CI 1.210–7.418, p = 0.018). When clinical characteristics of the patients were compared according to COMMD1 expression, no differences in gender, subtype, age, LDH level or stage were observed between the subgroups (Table 3). However, low COMMD1 expression was associated with low performance status.

We also examined prognostic impact on COMMD1 expression according to molecular subtype, and found that a significant adverse prognostic impact of COMMD1 expression was restricted to the GCB subgroup (Fig. 4D; p = 0.029). The relative risk of relapse according to COMMD1 expression within the GCB subgroup was 3.434 (95% CI 1.056–11.164, p = 0.040). Overall, immunohistochemically defined molecular subgroup was not associated with survival.

In contrast to the results from genomic and transcriptomic levels, no correlation was found between COMMD1 protein levels and CNA or gene expression data (r = 0.236). The observation suggests that post-transcriptional mechanisms may be involved in the regulation of COMMD1 protein levels in DLBCL.

### COMMD1 Expression in an Independent DLBCL Series

In order to further validate the importance of COMMD1 in DLBCL, we analysed the prognostic significance of COMMD1 expression in a larger independent cohort of 146 DLBCL patients treated with chemoimmunotherapy (Table 3). Median age of the whole cohort was 63 years (range, 16–84 years), median follow-up 64 months (range 20–133 months), predicted 5-year PFS 74%, lymphoma-specific OS 79%, and OS 71%. While high IPI score was a strong predictor for survival (p<0.001), immunohistochemically defined molecular subgroup was not associated with outcome.

The clinical features of the patients according to COMMD1 expression (low versus high, cut-off defined according to TMA cohort) are summarized in Table 3. Accordingly, no differences were observed between COMMD1 low and high subgroups. In Kaplan-Meier analyses, PFS at five years for the patients with high COMMD1 expression was 64% compared with 79% for those with lower expression levels (*p* = 0.034; Figure 4E). When adjusted for IPI, COMMD1 expression maintained its prognostic effect on PFS (*p* = 0.023). In addition, when adjusted for age (<60 vs. ≥60), COMMD1 expression remained predictor for PFS (*p* = 0.035). According to COMMD1 expression, the relative risk or relapse was 1.9 (95% CI 1.040–3.606, *p* = 0.037). In multivariate analysis with IPI, COMMD1 retained its prognostic value on PFS (RR 2.0; 95% CI 1.037–3.730, *p* = 0.038).

Finally, when adjusted for molecular subgroups, COMMD1 expression was marginally predictive for PFS (*p* = 0.066). When COMMD1-related PFS was analysed separately for the patients in different molecular subtypes, there was a non-significant difference in PFS between COMMD1 high and low subgroups in the GCB DLBCLs (Fig. 4F; *p* = NS). Collectively, the results in our two independent cohorts provide evidence that COMMD1 is a novel survival-associated marker in DLBCLs.

## Discussion

Although the addition of rituximab to chemotherapy has considerably improved the survival rates of DLBCL, the patients with high IPI scores still have a poor prognosis. Here we have studied genomic alterations and gene expression in freshly frozen lymphoma tissue collected prospectively from patients included in a Nordic phase II study for young high-risk DLBCL patients. With this comprehensive approach we have identified copy number gain at 2p15 driving *COMMD1* mRNA upregulation with impact on survival. The gain was more frequently seen in the GCB than ABC DLBCLs. Furthermore, we have validated the results at the protein level by IHC in the same patient cohort as well as in an independent larger series. These data show that COMMD1 is a novel biomarker candidate that may be useful in improving risk stratification for DLBCL patients.

In a previous prospective study of poor prognosis DLBCL patients treated uniformly with dose-escalated CHOP followed by high-dose chemotherapy with autologous stem cell transplantation, the amplification of 2p16.1 was suggested to be associated with poor treatment response [34]. Candidate genes mapped within this amplification include proto-oncogenes *REL* and *BCL11A*, with increased expression levels [31], [35]. However, the functional correlation of this amplification event has not been illustrated [36]. Although *REL* and *BCL11A* expression levels were integrated with chromosomal amplification in our cohort, they were not associated with survival. Instead, we observed that the increased copy number in region 2p15 was associated with adverse PFS and lymphoma-specific OS. The region covers five genes, *CCT4, XPO1, COMMD1*, *FAM161A* and *B3GNT2*, which are all potentially important regulators of cellular growth. None of the genes, however, have previously been associated with DLBCL biology. While further investigation of the roles of these genes in DLBCL pathogenesis is needed, our results demonstrate that *COMMD1* is a candidate genetic prognostic biomarker in DLBCL.

COMMD1 is a pleiotropic factor that participates in multiple processes, including copper metabolism, sodium excretion, inflammatory responses, and adaptation to hypoxia [37]. Recent mechanistic studies have revealed that COMMD1 suppresses NF-kB- and HIF-mediated gene expression [38], [39]. *COMMD1* is underexpressed in some carcinomas, and low *COMMD1* expression has been associated with inferior clinical outcome in patients with endometrial cancer [39]. In lymphomas, the prognostic role of COMMD1 has not previously been established. In the Oncomine database [40] the expression of *COMMD1* is increased in lymphomas in comparison to other cancers [41]. Furthermore, two independent studies showed that *COMMD1* expression is higher in DLBCLs than follicular lymphomas [41], [42], whereas no differences in the *COMMD1* expression were observed between molecular subtypes of DLBCL [9]. At the present time, it remains unclear how COMMD1 is involved in a variety of seemingly unrelated and even opposite cellular activities. However, in most instances including lymphomas, the mechanism is likely via protein-protein interactions and ubiquitination [39], [43], [44].

To confirm whether COMMD1 expression could be useful to recognize DLBCLs with a more aggressive clinical course, we studied COMMD1 protein expression by IHC, which is a method that can be easily incorporated into a routine diagnostic approach. The predictive value of COMMD1 positivity was first defined in a training cohort of clinically high-risk DLBCL patients, and subsequently confirmed in an independent, larger and more heterogeneous DLBCL cohort. Thus, COMMD1 expression seems to represent a potential novel prognostic marker preferentially in the GCB type molecular subgroup.

Recently Monti and colleagues investigated gene expression and copy number data in 168 DLBCL patients, with the focus on the role of p53/cell cycle pathway in patient survival [28]. Interestingly, even though they reported that the region harboring *COMMD1* is the second highest region in their cohort, and *COMMD1* among the top genes in the region (Table1S in their publication [28]), they did not study its survival association. Additionally, our findings are supported by reports showing amplification of 2p15-p16 with the concordant elevated gene expression in DLBCL [28], [31], [34], [35]. These indicate that while integration of copy number and expression data is known to be a powerful approach to find driver genes, carefully selected, homogenous patient cohort together with integrative analysis can produce clear and important findings that may not be evident in more heterogeneous cohorts.

Since CNA status was integrated with gene expression data, only the genes, whose expression correlated with the CNAs were identified. The results from the qRT-PCR also correlated with the exon array data. However, we found no correlation between COMMD1 protein and gene expression levels. The reason for this difference is currently unknown but based on the literature indicating a strong regulatory role for the processes downstream of transcription [45], [46], it is plausible to speculate that post-transcriptional mechanisms may have a role in the regulation of COMMD1 protein levels in DLBCL. The work demonstrating that COMMD1 cellular levels are tightly controlled by ubiquitination [47] provides additional evidence that the regulatory level may be posttranslational. Together with the CNA data the results indicate that COMMD1 expression is regulated at multiple levels.

Amplification in 18q12.2 was found to be another significant CNA associated with inferior outcome. The gain has not been previously associated with survival in DLBCL, but its deletion has been reported to correlate with poor outcome in colorectal carcinoma. The CNA was found to harbour a single gene, *CELF4/CUGBP*, coding for a member of a family of RNA binding proteins playing an essential role in post-transcriptional gene regulation. However, despite the gain in 18q12.2 locus we were not able to demonstrate the over-expression of the *CELF4* gene. While more work is needed to establish the exact role of the 18q12.2 gain in DLBCL, our data supports the prognostic importance of this region in DLBCL.

Consistent with previous studies [27], [30], [32], [33], expression of CDKN2A was associated with deletion of 9p21.3 in our patient cohort, and especially in the non-GCB subtype. However, overall incidence of the deletion was lower and no correlation with survival was found. Considering that aCGH and exon arrays were not performed on purified tumour samples it is possible that the presence of background material (tumour infiltrating non-malignant cells) could to some extent dilute tumour specific genetic alterations and explain a lower incidence of 9p21 deletions in our material. However, a more likely explanation for the differences in the results between different studies is that clinical and histopathological features of the study populations are not identical. The differences in the treatments may also contribute.

In conclusion, we have integrated copy number alteration and transcriptomic data in a carefully chosen high-risk DLBCL patient cohort to identify biological markers that could be used in risk stratification. We found two profiles with increased copy number of genes in chromosomes 2p15 and 18q12.2 that predicted a poor outcome for a subgroup of DLBCL patients. Furthermore, we identified a novel potential genetic driver event with prognostic significance. Notably, the prognostic impact of COMMD1 on survival was also observed at the protein level. The strengths of our study are a prospectively collected and homogenously treated study population, the availability of copy number, gene expression and IHC data from the same patients, the possibility to correlate the findings with clinical outcome, and validate the findings in an independent cohort of DLBCL patients. Our results demonstrate that it is possible to use relatively small but carefully designed prospective cohorts as a hypothesis generating material to identify a list of putative targets, and then validate and extend the major results to the protein level. Taken together, the results presented herein are promising and novel, and emphasize the importance of integrated genetic information and multilevel analyses for both the optimal use of existing combination therapies and the development of novel treatments for DLBCL.

## Supporting Information

File S1 Table S1, The most recurrent CNAs distributed between molecular subgroups. Table S2, Gene expression changes associated with CNAs. Figure S1, Association of *COMMD1* (A) and *XPO1* (B) overexpression with 2p15 amplification according to qRT-PCR analysis based expression values.
(PDF)

## References

[1] Coiffier B, Lepage E, Briere J, Herbrecht R, Tilly H, et al (2002) CHOP chemotherapy plus rituximab compared with CHOP alone in elderly patients with diffuse large-B-cell lymphoma. N Engl J Med 346: 235–242. 11807147 10.1056/NEJMoa011795

[2] Habermann TM, Weller EA, Morrison VA, Gascoyne RD, Cassileth PA, et al (2006) Rituximab-CHOP versus CHOP alone or with maintenance rituximab in older patients with diffuse large B-cell lymphoma. J Clin Oncol 24: 3121–3127. 16754935 10.1200/JCO.2005.05.1003

[3] Pfreundschuh M, Schubert J, Ziepert M, Schmits R, Mohren M, et al (2008) Six versus eight cycles of bi-weekly CHOP-14 with or without rituximab in elderly patients with aggressive CD20+ B-cell lymphomas: a randomised controlled trial (RICOVER-60). Lancet Oncol 9: 105–116. 18226581 10.1016/S1470-2045(08)70002-0

[4] Pfreundschuh M, Trumper L, Kloess M, Schmits R, Feller AC, et al (2004) Two-weekly or 3-weekly CHOP chemotherapy with or without etoposide for the treatment of young patients with good-prognosis (normal LDH) aggressive lymphomas: results of the NHL-B1 trial of the DSHNHL. Blood 104: 626–633. 14982884 10.1182/blood-2003-06-2094

[5] Pfreundschuh M, Trumper L, Osterborg A, Pettengell R, Trneny M, et al (2006) CHOP-like chemotherapy plus rituximab versus CHOP-like chemotherapy alone in young patients with good-prognosis diffuse large-B-cell lymphoma: a randomised controlled trial by the MabThera International Trial (MInT) Group. Lancet Oncol 7: 379–391. 16648042 10.1016/S1470-2045(06)70664-7

[6] Lenz G, Staudt LM (2010) Aggressive lymphomas. N Engl J Med 362: 1417–1429. 20393178 10.1056/NEJMra0807082 PMC7316377

[7] Shaffer AL 3rd, Young RM, Staudt LM (2012) Pathogenesis of human B cell lymphomas. Annu Rev Immunol 30: 565–610. 22224767 10.1146/annurev-immunol-020711-075027 PMC7478144

[8] Alizadeh AA, Eisen MB, Davis RE, Ma C, Lossos IS, et al (2000) Distinct types of diffuse large B-cell lymphoma identified by gene expression profiling. Nature 403: 503–511. 10676951 10.1038/35000501

[9] Lenz G, Wright G, Dave SS, Xiao W, Powell J, et al (2008) Stromal gene signatures in large-B-cell lymphomas. N Engl J Med 359: 2313–2323. 19038878 10.1056/NEJMoa0802885 PMC9103713

[10] Rosenwald A, Wright G, Chan WC, Connors JM, Campo E, et al (2002) The use of molecular profiling to predict survival after chemotherapy for diffuse large-B-cell lymphoma. N Engl J Med 346: 1937–1947. 12075054 10.1056/NEJMoa012914

[11] Rosenwald A, Wright G, Leroy K, Yu X, Gaulard P, et al (2003) Molecular diagnosis of primary mediastinal B cell lymphoma identifies a clinically favorable subgroup of diffuse large B cell lymphoma related to Hodgkin lymphoma. J Exp Med 198: 851–862. 12975453 10.1084/jem.20031074 PMC2194208

[12] Huang JZ, Sanger WG, Greiner TC, Staudt LM, Weisenburger DD, et al (2002) The t(14;18) defines a unique subset of diffuse large B-cell lymphoma with a germinal center B-cell gene expression profile. Blood 99: 2285–2290. 11895757 10.1182/blood.v99.7.2285

[13] Davis RE, Brown KD, Siebenlist U, Staudt LM (2001) Constitutive nuclear factor kappaB activity is required for survival of activated B cell-like diffuse large B cell lymphoma cells. J Exp Med 194: 1861–1874. 11748286 10.1084/jem.194.12.1861 PMC2193582

[14] Lohr JG, Stojanov P, Lawrence MS, Auclair D, Chapuy B, et al (2012) Discovery and prioritization of somatic mutations in diffuse large B-cell lymphoma (DLBCL) by whole-exome sequencing. Proc Natl Acad Sci U S A 109: 3879–3884. 22343534 10.1073/pnas.1121343109 PMC3309757

[15] Morin RD, Mendez-Lago M, Mungall AJ, Goya R, Mungall KL, et al (2011) Frequent mutation of histone-modifying genes in non-Hodgkin lymphoma. Nature 476: 298–303. 21796119 10.1038/nature10351 PMC3210554

[16] Morin RD, Mungall K, Pleasance E, Mungall AJ, Goya R, et al (2013) Mutational and structural analysis of diffuse large B-cell lymphoma using whole-genome sequencing. Blood 122: 1256–1265. 23699601 10.1182/blood-2013-02-483727 PMC3744992

[17] Pasqualucci L, Trifonov V, Fabbri G, Ma J, Rossi D, et al (2011) Analysis of the coding genome of diffuse large B-cell lymphoma. Nat Genet 43: 830–837. 21804550 10.1038/ng.892 PMC3297422

[18] Holte H, Leppa S, Bjorkholm M, Fluge O, Jyrkkio S, et al (2013) Dose-densified chemoimmunotherapy followed by systemic central nervous system prophylaxis for younger high-risk diffuse large B-cell/follicular grade 3 lymphoma patients: results of a phase II Nordic Lymphoma Group study. Ann Oncol 24: 1385–1392. 23247661 10.1093/annonc/mds621

[19] Swerdlow SH, Campo E, Harris NL, Jaffe E, Pileri A, et al.. (2008) WHO Classification of tumours of hematopoietic and lymphoid tissues. Lyon: IARCH Press.

[20] Wright G, Tan B, Rosenwald A, Hurt EH, Wiestner A, et al (2003) A gene expression-based method to diagnose clinically distinct subgroups of diffuse large B cell lymphoma. Proc Natl Acad Sci U S A 100: 9991–9996. 12900505 10.1073/pnas.1732008100 PMC187912

[21] Hans CP, Weisenburger DD, Greiner TC, Gascoyne RD, Delabie J, et al (2004) Confirmation of the molecular classification of diffuse large B-cell lymphoma by immunohistochemistry using a tissue microarray. Blood 103: 275–282. 14504078 10.1182/blood-2003-05-1545

[22] Ovaska K, Laakso M, Haapa-Paananen S, Louhimo R, Chen P, et al (2010) Large-scale data integration framework provides a comprehensive view on glioblastoma multiforme. Genome Med 2: 65. 20822536 10.1186/gm186 PMC3092116

[23] Olshen AB, Venkatraman ES, Lucito R, Wigler M (2004) Circular binary segmentation for the analysis of array-based DNA copy number data. Biostatistics 5: 557–572. 15475419 10.1093/biostatistics/kxh008

[24] Iafrate AJ, Feuk L, Rivera MN, Listewnik ML, Donahoe PK, et al (2004) Detection of large-scale variation in the human genome. Nat Genet 36: 949–951. 15286789 10.1038/ng1416

[25] Chen P, Lepikhova T, Hu Y, Monni O, Hautaniemi S (2011) Comprehensive exon array data processing method for quantitative analysis of alternative spliced variants. Nucleic Acids Res 39: e123. 21745820 10.1093/nar/gkr513 PMC3185423

[26] Hyman E, Kauraniemi P, Hautaniemi S, Wolf M, Mousses S, et al (2002) Impact of DNA amplification on gene expression patterns in breast cancer. Cancer Res 62: 6240–6245. 12414653

[27] Lenz G, Wright GW, Emre NC, Kohlhammer H, Dave SS, et al (2008) Molecular subtypes of diffuse large B-cell lymphoma arise by distinct genetic pathways. Proc Natl Acad Sci U S A 105: 13520–13525. 18765795 10.1073/pnas.0804295105 PMC2533222

[28] Monti S, Chapuy B, Takeyama K, Rodig SJ, Hao Y, et al (2012) Integrative analysis reveals an outcome-associated and targetable pattern of p53 and cell cycle deregulation in diffuse large B cell lymphoma. Cancer Cell 22: 359–372. 22975378 10.1016/j.ccr.2012.07.014 PMC3778921

[29] Tagawa H, Suguro M, Tsuzuki S, Matsuo K, Karnan S, et al (2005) Comparison of genome profiles for identification of distinct subgroups of diffuse large B-cell lymphoma. Blood 106: 1770–1777. 15886317 10.1182/blood-2005-02-0542

[30] Takeuchi I, Tagawa H, Tsujikawa A, Nakagawa M, Katayama-Suguro M, et al (2009) The potential of copy number gains and losses, detected by array-based comparative genomic hybridization, for computational differential diagnosis of B-cell lymphomas and genetic regions involved in lymphomagenesis. Haematologica 94: 61–69. 19029149 10.3324/haematol.12986 PMC2625430

[31] Bea S, Zettl A, Wright G, Salaverria I, Jehn P, et al (2005) Diffuse large B-cell lymphoma subgroups have distinct genetic profiles that influence tumor biology and improve gene-expression-based survival prediction. Blood 106: 3183–3190. 16046532 10.1182/blood-2005-04-1399 PMC1895326

[32] Kreisel F, Kulkarni S, Kerns RT, Hassan A, Deshmukh H, et al (2011) High resolution array comparative genomic hybridization identifies copy number alterations in diffuse large B-cell lymphoma that predict response to immuno-chemotherapy. Cancer Genet 204: 129–137. 21504712 10.1016/j.cancergen.2010.12.010 PMC3837631

[33] Jardin F, Jais JP, Molina TJ, Parmentier F, Picquenot JM, et al (2010) Diffuse large B-cell lymphomas with CDKN2A deletion have a distinct gene expression signature and a poor prognosis under R-CHOP treatment: a GELA study. Blood 116: 1092–1104. 20435884 10.1182/blood-2009-10-247122

[34] Robledo C, Garcia JL, Caballero D, Conde E, Arranz R, et al (2009) Array comparative genomic hybridization identifies genetic regions associated with outcome in aggressive diffuse large B-cell lymphomas. Cancer 115: 3728–3737. 19517471 10.1002/cncr.24430

[35] Fukuhara N, Tagawa H, Kameoka Y, Kasugai Y, Karnan S, et al (2006) Characterization of target genes at the 2p15–16 amplicon in diffuse large B-cell lymphoma. Cancer Sci 97: 499–504. 16734728 10.1111/j.1349-7006.2006.00209.x PMC11159140

[36] Houldsworth J, Olshen AB, Cattoretti G, Donnelly GB, Teruya-Feldstein J, et al (2004) Relationship between REL amplification, REL function, and clinical and biologic features in diffuse large B-cell lymphomas. Blood 103: 1862–1868. 14615382 10.1182/blood-2003-04-1359

[37] Maine GN, Burstein E (2007) COMMD proteins: COMMing to the scene. Cell Mol Life Sci 64: 1997–2005. 17497243 10.1007/s00018-007-7078-y PMC2938186

[38] Maine GN, Burstein E (2007) COMMD proteins and the control of the NF kappa B pathway. Cell Cycle 6: 672–676. 17361106 10.4161/cc.6.6.3989 PMC2910620

[39] van de Sluis B, Mao X, Zhai Y, Groot AJ, Vermeulen JF, et al (2010) COMMD1 disrupts HIF-1alpha/beta dimerization and inhibits human tumor cell invasion. J Clin Invest 120: 2119–2130. 20458141 10.1172/JCI40583 PMC2877941

[40] Rhodes DR, Yu J, Shanker K, Deshpande N, Varambally R, et al (2004) Large-scale meta-analysis of cancer microarray data identifies common transcriptional profiles of neoplastic transformation and progression. Proc Natl Acad Sci U S A 101: 9309–9314. 15184677 10.1073/pnas.0401994101 PMC438973

[41] Ramaswamy S, Tamayo P, Rifkin R, Mukherjee S, Yeang CH, et al (2001) Multiclass cancer diagnosis using tumor gene expression signatures. Proc Natl Acad Sci U S A 98: 15149–15154. 11742071 10.1073/pnas.211566398 PMC64998

[42] Shipp MA, Ross KN, Tamayo P, Weng AP, Kutok JL, et al (2002) Diffuse large B-cell lymphoma outcome prediction by gene-expression profiling and supervised machine learning. Nat Med 8: 68–74. 11786909 10.1038/nm0102-68

[43] Drevillon L, Tanguy G, Hinzpeter A, Arous N, de Becdelievre A, et al (2011) COMMD1-mediated ubiquitination regulates CFTR trafficking. PLoS One 6: e18334. 21483833 10.1371/journal.pone.0018334 PMC3069076

[44] Thoms HC, Loveridge CJ, Simpson J, Clipson A, Reinhardt K, et al (2010) Nucleolar targeting of RelA(p65) is regulated by COMMD1-dependent ubiquitination. Cancer Res 70: 139–149. 20048074 10.1158/0008-5472.CAN-09-1397

[45] Schwanhausser B, Busse D, Li N, Dittmar G, Schuchhardt J, et al (2011) Global quantification of mammalian gene expression control. Nature 473: 337–342. 21593866 10.1038/nature10098

[46] Vogel C, Marcotte EM (2012) Insights into the regulation of protein abundance from proteomic and transcriptomic analyses. Nat Rev Genet 13: 227–232. 22411467 10.1038/nrg3185 PMC3654667

[47] Maine GN, Mao X, Muller PA, Komarck CM, Klomp LW, et al (2009) COMMD1 expression is controlled by critical residues that determine XIAP binding. Biochem J 417: 601–609. 18795889 10.1042/BJ20080854 PMC2606926

