# Supplementary material for: Deregulation of COMMD1 Is Associated with Poor Prognosis in Diffuse Large B-cell Lymphoma
Source: PLoS One. 2014 Mar 13;9(3):e91031. doi: 10.1371/journal.pone.0091031 (PMC3953211; doi:10.1371/journal.pone.0091031)

Supplementary Material

Deregulation of COMMD1 is associated with poor prognosis in diffuse large B-cell lymphoma

Minna Taskinen[1,2], Riku Louhimo [2], Satu Koivula[1,2], Ping Chen[2], Ville Rantanen[2], Harald Holte[3], Jan Delabie[4], Marja-Liisa Karjalainen-Lindsberg[5], Magnus Björkholm[6], Øystein Fluge[7], Lars Møller Pedersen[8**], Karin Fjordén[9], Mats Jerkeman[9], Mikael Eriksson[9], Sampsa Hautaniemi[2] and Sirpa Leppä[1,2]

[1]Department of Oncology, Helsinki University Central Hospital, Helsinki, Finland; [2]Genome Scale Biology Program, University of Helsinki, Helsinki, Finland; [3]Department of Oncology, Oslo University Hospital, Oslo, Norway; [4]Department of Pathology, Oslo University Hospital, Oslo, Norway; [5]Department of Pathology, Haartman Institute, University of Helsinki, Helsinki, Finland; [6]Department of Medicine, Karolinska University Hospital, Stockholm, Sweden; [7]Department of Oncology and Medical Physics, Haukeland University Hospital, Bergen, Norway; [8]Department of Hematology, Odense University Hospital, Odense, Denmark; [9]Department of Oncology, Lund University Hospital, Lund, Sweden

***Present address: Department of Hematology, Roskilde University Hospital, Roskilde, Denmark

Table S1. The most recurrent CNAs distributed between molecular subgroups

Gain	All n (%) n=51	Non-GCB, n (%) n=20	GCB, n (%) n=27	Gene	Gene Description	CNV
1q24.2	6 (12)	3 (15)	3 (11)	MPZL1	myelin protein zero-like 1	0
1q44	10 (20)	4 (20)	6 (22)	AHCTF1	AT hook containing transcription factor 1	1
2p16.1	6 (12)	1 (5)	5 (19)	BCL11A	B-cell CLL/lymphoma 11A (zinc finger protein)	4
				PAPOLG	poly(A) polymerase gamma	3
				AC010733.4	[undefined]	3
				REL	v-rel reticuloendotheliosis viral oncogene homolog (avian)	3
				PUS10	pseudouridylate synthase 10	3
				PEX13	peroxisomal biogenesis factor 13	3
2p15, 2p16.1	6 (12)	1 (5)	5 (19)	KIAA1841	KIAA1841	3
2p15	6 (12)	1 (5)	5 (19)	AC016747.3	[undefined]	0
				AHSA2	AHA1, activator of heat shock 90kDa protein ATPase homolog 2	0
				USP34	ubiquitin specific peptidase 34	1
	6 (12)	1 (5)	4 (15)	XPO1	exportin 1 (CRM1 homolog)	1
				FAM161A	family with sequence similarity 161, member A	1
				CCT4	chaperonin containing TCP1, subunit 4 (delta)	1
				COMMD1	copper metabolism (Murr1) domain containing 1	1
				AC018462.2	[undefined]	1
				B3GNT2	UDP-GlcNAc:betaGal beta-1,3-N-acetylglucosaminyltransferase 2	0
				TMEM17	transmembrane protein 17	0
				AC092155.4	[undefined]	1
				EHBP1	EH domain binding protein 1	0
18q12.2	6 (12)	4 (20)	1 (4)	CELF4	CUGBP, Elav-like family member 4	1
18q21.1	6 (12)	2 (10)	3 (11)	LOXHD1	lipoxygenase homology domains 1	1
				ST8SIA5	ST8 alpha-N-acetyl-neuraminide alpha-2,8-sialyltransferase 5	1
18q23	8 (16)	4 (20)	2 (7)	KCNG2	potassium voltage-gated channel, subfamily G, member 2	1
	6 (12)	4 (20)	1 (4)	PQLC1	PQ loop repeat containing 1	0

20q11.22	8 (16)	5 (25)	3 (11)	MAP1LC3A	microtubule-associated protein 1 light chain 3 alpha	0
**Loss**	**All n (%) n=51**	**Non-GCB, n (%) n=20**	**GCB, n (%) n=27**	**Gene**	**Gene Description**	**CNV**
9p21.3	9 (18)	7 (35)	2 (7)	MTAP	methylthioadenosine phosphorylase	1
				CDKN2A	cyclin-dependent kinase inhibitor 2A	6
	8 (16)	6 (30)	2 (7)	CDKN2B-AS1	CDKN2B antisense RNA 1 (non-protein coding)	6
				CDKN2B	cyclin-dependent kinase inhibitor 2B	5
14q11.2	6 (12)	1 (5)	4 (15)	TRDV3	T cell receptor delta variable 3	10

GCB, germinal centre B, CNV, copy number variants

Table S2. Gene expression changes associated with CNAs.

Gene Id	Gene	Integration p value (Amp)	Gene description	Band
ENSG00000173209	AHSA2	0.00	AHA1, activator of heat shock 90kDa protein ATPase homolog 2 (yeast)	2p15
ENSG00000115464	USP34	0.00	ubiquitin specific peptidase 34	2p15
ENSG00000082898	XPO1	0.00	exportin 1 (CRM1 homolog, yeast)	2p15
ENSG00000170264	FAM161A	0.01	family with sequence similarity 161, member A	2p15
ENSG00000115484	CCT4	0.00	chaperonin containing TCP1, subunit 4 (delta)	2p15
ENSG00000173163	COMMD1	0.00	copper metabolism (Murr1) domain containing 1	2p15
ENSG00000170340	B3GNT2	0.02	UDP-GlcNAc:betaGal beta-1,3-N-acetylglucosaminyltransferase 2	2p15
ENSG00000162929	KIAA1841	0.00	KIAA1841	2p15,2p16.1
ENSG00000119866	BCL11A	0.00	B-cell CLL/lymphoma 11A (zinc finger protein)	2p16.1
ENSG00000115421	PAPOLG	0.00	poly(A) polymerase gamma	2p16.1
ENSG00000162924	REL	0.00	v-rel reticuloendotheliosis viral oncogene homolog (avian)	2p16.1
ENSG00000162927	PUS10	0.00	pseudouridylate synthase 10	2p16.1
ENSG00000162928	PEX13	0.00	peroxisomal biogenesis factor 13	2p16.1
ENSG00000196628	TCF4	0.00	transcription factor 4	18q21.2

ENSG00000091164	TXNL1	0.00	thioredoxin-like 1	18q21.31
ENSG00000091157	WDR7	0.00	WD repeat domain 7	18q21.31
ENSG00000177511	ST8SIA3	0.01	ST8 alpha-N-acetyl-neuraminide alpha-2,8-sialyltransferase 3	18q21.31
ENSG00000066926	FECH	0.00	ferrochelatase	18q21.31
ENSG00000134440	NARS	0.00	asparaginyl-tRNA synthetase	18q21.31
ENSG00000172175	MALT1	0.03	mucosa associated lymphoid tissue lymphoma translocation gene 1	18q21.32
ENSG00000074657	ZNF532	0.00	zinc finger protein 532	18q21.32
ENSG00000074695	LMAN1	0.00	lectin, mannose-binding, 1	18q21.32
ENSG00000141682	PMAIP1	0.02	phorbol-12-myristate-13-acetate-induced protein 1	18q21.32
ENSG00000166603	MC4R	0.02	melanocortin 4 receptor	18q21.32
ENSG00000197563	PIGN	0.00	phosphatidylinositol glycan anchor biosynthesis, class N	18q21.33
ENSG00000134444	KIAA1468	0.00	KIAA1468	18q21.33
ENSG00000141664	ZCCHC2	0.00	zinc finger, CCHC domain containing 2	18q21.33
ENSG00000171791	BCL2	0.00	B-cell CLL/lymphoma 2	18q21.33
ENSG00000122490	PQLC1	0.00	PQ loop repeat containing 1	18q23

Gene Id	Gene	Integration p value (Del)	Gene description	Band
ENSG00000099810	MTAP	0.00	methylthioadenosine phosphorylase	9p21.3
ENSG00000147889	CDKN2A	0.03	cyclin-dependent kinase inhibitor 2A (melanoma, p16, inhibits CDK4)	9p21.3

Figure S1. Association of *COMMD1* (A) and *XPO1* (B) overexpression with 2p15 amplification according to qRT-PCR analysis based expression values.

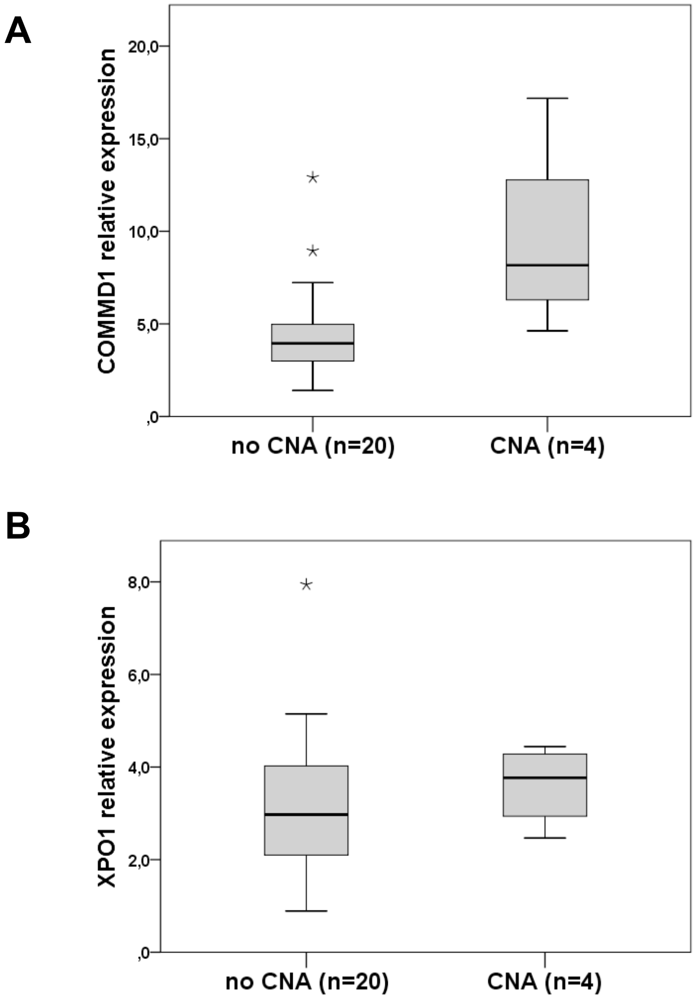

Supplement: File S1 — Table S1, The most recurrent CNAs distributed between molecular subgroups. Table S2, Gene expression changes associated with CNAs. Figure S1, Association of COMMD1 (A) and XPO1 (B) overexpression with 2p15 amplification according to qRT-PCR analysis based expression values. (PDF) [file pone.0091031.s001.pdf]
